# Deep learning for tubes and lines detection in critical illness: Generalizability and comparison with residents

**DOI:** 10.1016/j.ejro.2024.100593

**Published:** 2024-07-29

**Authors:** Pootipong Wongveerasin, Trongtum Tongdee, Pairash Saiviroonporn

**Affiliations:** Department of Radiology, Faculty of Medicine Siriraj Hospital, Mahidol University, Bangkok, Thailand

**Keywords:** Tubes and lines, chest radiograph, artificial intelligence, deep learning, generalizability, residents

## Abstract

**Background:**

Artificial intelligence (AI) has been proven useful for the assessment of tubes and lines on chest radiographs of general patients. However, validation on intensive care unit (ICU) patients remains imperative.

**Methods:**

This retrospective case-control study evaluated the performance of deep learning (DL) models for tubes and lines classification on both an external public dataset and a local dataset comprising 303 films randomly sampled from the ICU database. The endotracheal tubes (ETTs), central venous catheters (CVCs), and nasogastric tubes (NGTs) were classified into “Normal,” “Abnormal,” or “Borderline” positions by DL models with and without rule-based modification. Their performance was evaluated using an experienced radiologist as the standard reference.

**Results:**

The algorithm showed decreased performance on the local ICU dataset, compared to that of the external dataset, decreasing from the Area Under the Curve of Receiver (AUC) of 0.967 (95 % CI 0.965–0.973) to the AUC of 0.70 (95 % CI 0.68–0.77). Significant improvement in the ETT classification task was observed after modifications were made to the model to allow the use of the spatial relationship between line tips and reference anatomy with the improvement of the AUC, increasing from 0.71 (95 % CI 0.70 – 0.75) to 0.86 (95 % CI 0.83 – 0.94)

**Conclusions:**

The externally trained model exhibited limited generalizability on the local ICU dataset. Therefore, evaluating the performance of externally trained AI before integrating it into critical care routine is crucial. Rule-based algorithm may be used in combination with DL to improve results.

## 1 Background

With the increasing clinical application of artificial intelligence (AI) in patient care, AI-assisted tubes and lines assessment on chest radiographs (CXR) has become a hot topic in recent years [1], [2], [3], [4], [5], since misplaced tubes, such as endotracheal tube (ETT), found in up to 5–28 % of intubated patients, can result in asphyxia, desaturation, or even death [6].

ABM Khan et al., winner of the tubes and lines classification competition hosted by the Royal Australian and New Zealand College of Radiologists (RANZCR) in 2021[5], created a deep learning (DL) model that classifies the positions of ETTs, central venous catheters (CVCs), and nasogastric tubes (NGTs) into “Normal”, “Abnormal”, or “Borderline”. This experimental AI system could prove helpful in critical care settings since it could cover the three common tubes and lines present in ICU patients. However, as highlighted by Saqib et al., the clinical application of AI in intensive care unit (ICU) patients, who are particularly vulnerable, is hindered by a lack of sufficient clinical trials and, consequentially, low reproducibility [7]. A recent systematic review showed that the vast majority (70 of 86, 81 %) of algorithms reported some decrease in external performance compared to internal performance [8], [9], [10]. Since most previous works were trained and evaluated on public datasets [3], [5], [11], [12], our study aims to evaluate the state-of-the-art model specifically on our local ICU dataset. This task is challenging because ICU patients often have more advanced diseases, resulting in a greater number of lines to be interpreted per film. Additionally, the extensive disease itself may be present in the film, which can complicate interpretation.

Furthermore, to tackle the “Black box” problem of the original model, we made slight modifications to the original model by separating each tube and line into their own channels. This modification not only allowed for color coding of each line provided better visual aid for physicians but also enabled reporting of distances between the ETT and the carina (ETT-carina distance) and between the CVC and the cavoatrial junction (CVC-cavoatrial junction distance). This is especially pertinent for ICU patients since we aimed to create a system that not only enhances interpretability but also provides essential metrics for clinical decision-making.

By training the model on an external dataset, as in the original work, and testing it on the local ICU population, we mimic the development process of commercially available models. This approach allows us to further evaluate the problem of limited generalizability, modify the model to better suit our use case, and describe the results and insights as gathered.

## 2 Methods

### 2.1 Methods

This retrospective case-control study was conducted from Jan 2022 to Jan 2023 at a tertiary-care urban academic hospital with a capacity of 2200 beds. The hospital’s Institutional Review Board approved the study (ID: 843/2565(IRB2)) and consent was waived due to its retrospective nature. The dataset produced from this work was anonymized according to the hospital’s standard procedures.

### 2.2 Algorithm

In 2021, RANZCR hosted a competition for tube and line classification [13]. The objective of the competition was to develop models capable of accurately classifying the CXR images depicting ETT, CVC, and NGT into “Normal”, “Abnormal”, and “Borderline” categories. The winning algorithm by ABM Khan et al. consists of a series of models heavily based on UNET++ and EfficientNet [5]. The smaller model (EfficientNet-B1) achieved an average Area Under the Curve (AUC) score of 0.972, which was not significantly different from the larger model (EfficientNet-B5) at 0.973 but consumed fewer resources. Thus, the smaller model was used in this study, hereafter referred to as the ‘original model'.

This model employs a two-step approach. The first step, termed the segmentation step, involves the segmentation of the tubes and lines, generating two mask channels: the line segmentation mask and the tip segmentation mask. The model used in this step is named ‘segmentation model 1’. Additionally, the carina position was also segmented using ‘Segmentation model 2’. The second step is the classification step, which takes the input image along with the two channels of masks created in step 1 as input and classifies them using a multiclass classifier based on RANZCR – CLiP definitions shown in Table 1 [13].

**Table 1:** Criteria for tubes and lines classification based on RANZCR – CLiP definitions [13].

Tubes/Lines tips	Normal	Borderline	Abnormal	Incompletely imaged
Endotracheal Tubes (ETT)	Above the upper margin of the aortic arch or two posterior ribs^†^ above the carina*	Below the aortic arch or less than two posterior ribs^†^ above the carina* or higher than two times two posterior ribs^†^ above the carina*	At or below the carina or at level of T1 or above.	N/A
Nasogastric tubes (NGT)	10 cm from gastroesophageal junction (measured using “Four posterior ribs”^‡^)	Beyond, but less than 10 cm from the gastro-esophageal junction.	Within tracheobronchopulmonary system or above gastro-esophageal junction.	The NGT tip was outside the field of the radiograph and the remaining length within the radiograph is <10 cm.
Central venous catheters (CVC)	Over the SVC, below the upper margin of the aortic arch and above the cavoatrial junction**. The catheter also had to form an angle of less than 45 degrees with the vessel wall.	Proximal to the SVC, within the SVC but with an angle to the vessel wall of >45 degrees or below the cavoatrial junction** but with the tip remaining in the upper 1/3 of the right atrium	Below the upper 1/3 of the right atrium, coiled or kinked. Atypical position, such as with the tip in the azygos vein or internal thoracic vein or in the aorta or extravascular structures.	N/A
Swan-Ganz Catheters	All Swan-Ganz Catheters are automatically labeled as normal because it is difficult to delineate where the CVC sheath stops.			N/A

Gastroesophageal junction = Medial border of the left hemidiaphragm

†Two posterior ribs = Distance between the superior margin of one rib to the inferior margin of the rib below (∼3.5 cm).

‡Four posterior ribs = Distance between the superior margin of one rib to the inferior margin of the third tib (∼10 cm).

* Carina = Intersection of the inferior wall of right and left main bronchi

** Cavoatrial junction = Intersection of right heart border with inferior margin of bronchus intermedius or if the bronchus was not visible, labelers are advised to use the curvature of SVC contour and right heart border.

**Fig. 1:**
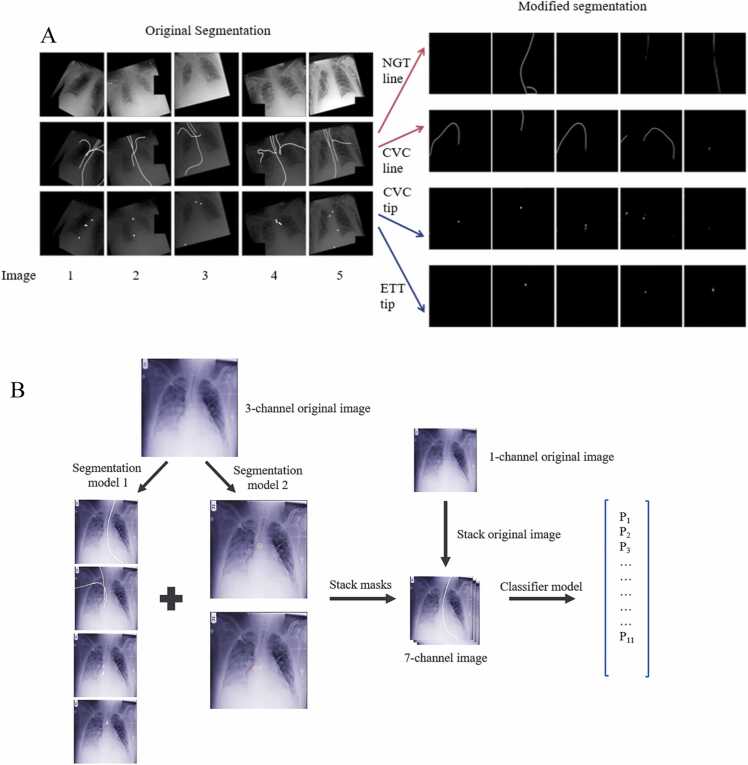
Original algorithm segmentation and modified algorithm segmentation. (A) The left side of the image shows the segmentation performed by the original algorithm. Each column represents a different input image (Image 1–5). The first row shows the images after augmentation according to the original paper. In the second and third rows, the line segmentations and tip segmentations are depicted, respectively, without being separated by class. The image on the right illustrates the segmentations produced by the modified algorithm, which includes more rows, with each row representing a single class in the following order from top to bottom: NGT line, CVC line, CVC tip, and ETT tip. (B) The resulting workflow of the two-stage approach of segmentation and classification.

This can be further improved by separating the masks according to their classes into “NGT line”, “CVC line”, “CVC tip”, and “ETT tip”, accordingly, as shown in Fig. 1. This modification will be referred to as ‘the modified model’. Please note that the 'ETT line' and 'NGT tip' were omitted because our preliminary experiments did not show a performance difference. The separation of masks allowed for the extraction of the tip coordinates using the Connected Component Algorithm from the OpenCV library [14].The tip coordinates can then be used to calculate the distance between the tip of the tubes and lines to their respective reference anatomy, namely, the carina and the cavoatrial junction. Subsequently, a rule-based system can be employed to further correct the output according to the rules outlined in Table 1, e.g., in the case of Borderline ETT, if the tip is less than 3.5 cm above the carina or more than 7 cm above the carina, or if the CVC tip is below the cavoatrial junction, it should be upgraded from “Normal” to “Borderline”. This will be called ‘the modified algorithm with rule-based system’.

### 2.3 Training and testing

**Fig. 2:**
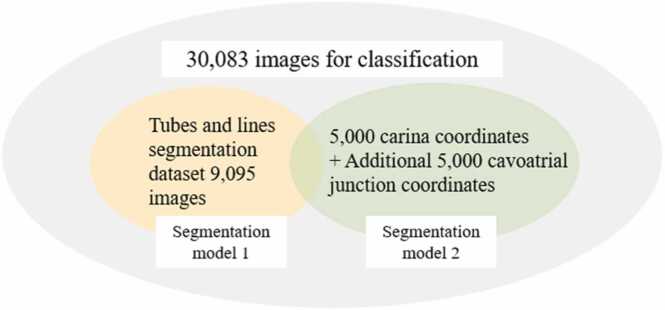
Dataset Composition for Classification and Segmentation Tasks. Out of a total of 30,083 images, 9095 images were used for tube and line segmentation. Another 5000 images were used for carina and cavoatrial junction segmentations. All 30,083 images were used in classification.

The dataset released by RANZCR consists of 30,083 CXR used in the original paper [5]. Of the 30,083 images, only a subset of 9083 images had segmentation data that could be used to train the segmentation model 1, and another subset of 5000 images contained carina coordinates and no cavoatrial junction coordinates. In order to make use of the cavoatrial junction-CVC distance, a new subset of randomly selected 5000 images were labeled with both carina and cavoatrial junction coordinates and were used in the training of the segmentation model 2. For the classification model, the entire dataset was used, as illustrated in Fig. 2. The training, validation, and testing dataset proportions were randomly split in an 80:10:10 ratio.

Both the original model and the modified model were trained on these external datasets using the PyTorch framework, following the parameters described in the original paper [5], employing ImageNet encoder weights [15], Albumentations for augementations [16], and utilized a learning rate scheme that included the Cosine Annealing method [17], A GradualWarmupScheduler and the Adam optimizer, and lastly, BCE with Logits Loss provided in the PyTorch framework [18]. The segmentation models used a learning rate of 1e-4, while the classification model used 3e-5, and all models were trained for 30 epochs. However, a 5-fold ensemble technique was not implemented due to its impracticality outside of competitive settings, as it would require significantly more computations for only marginal improvements in results.

Subsequently, the original model was tested on the same external dataset as a quality control measure and together with the modified models, it was then tested once more on the ‘local dataset’. The size of the local dataset was calculated by using MedCalc for the AUCs. Given that the original model has an AUC score of 0.972, the estimated percentage of abnormal tube and line positions is around 5–28 %, according to the study by Yi et al.[1]. The following parameters were used: an AUC of 0.9, a null hypothesis value of 0.7, and an estimated percentage of abnormal cases of 5 %, with alpha and beta of 0.05 and 0.2, respectively. The estimated total sample size was determined to be 300 cases. Therefore, 307 patients admitted to the adult ICUs during January 2023 were included in the study. However, four patients were excluded because they were under 18 years old. The local dataset was made by randomly selecting one radiograph DICOM file from each of the remaining 303 adult patients. The PNG images and metadata were extracted from the DICOM files, with windowing and rescaling applied using the Pydicom library [19]. The images were then labeled using the CVAT program [20]. The labeling of ground truth was performed by a cardiothoracic radiologist with 20+ years of experience without AI assistance.

**Fig. 3:**
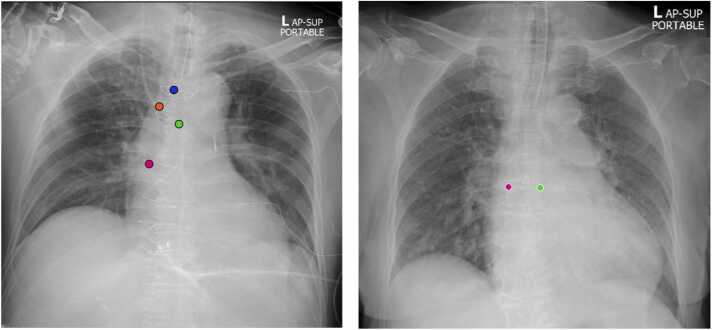
(A) AI-assisted labeling in CVAT: Normal tip position of ETT (blue dot), the borderline position of CVC tip (red dot), carina (green dot), and cavoatrial junction (pink dot) were represented. The users can freely adjust the position of each coordinate if needed. (B) Manual labeling, the coordinates of the carina (green dot) and cavoatrial junctions (pink dot) were placed near the center of the image, awaiting user adjustment. The tubes and lines coordinate instances must be created and placed manually.

For clinical evaluation against the residents, two senior residents (referred to by initials W. and V.) independently interpreted the radiographs twice: once with AI assistance using the modified algorithm and once without, with a one-month interval between each interpretation to prevent memory retention [21]. Before labeling, all participants received instructions on how to label using CVAT (Fig. 3 A) and the RANZCR criteria (Table 1). They underwent training with 30 mock images from the training dataset, ensuring that they placed labels at the tip of each tube and line to capture both the class and coordinates. For AI-assisted labeling, the labels were already placed on the images. In non-AI-assisted labeling, only the coordinates of the carina and cavoatrial junctions, present in all patients, are automatically placed near the center of the image, using CVAT’s propagate function (Fig. 3B).

### 2.4 Outcomes

The study aimed to evaluate the original model’s ability to classify tube and line positions in a local dataset comprising ICU patients, with primary outcomes measured by AUCs. Secondary outcomes included comparing the performance of the original model to that of the modified AI models, both with and without a rule-based system, as well as comparing the performance of the models with that of two senior residents. Additionally, the study assessed the interpretation time of both residents with and without AI assistance, evaluated the mean absolute error (MAE) of AI- and residents-labeled coordinates of the carina and cavoatrial junction compared to ground truth, and investigated the effects of other lines present in radiographs, namely, ECG leads, on the algorithm's classification performance.

### 2.5 Statistical analysis

Continuous variables were presented as means or medians and standard deviations (SD) or Interquartile range (IQR). Paired Samples T-Tests using SciPy v1.11.4 [22] were conducted to compare the distances of resident-labeled coordinates to those of radiologist-labeled coordinates of the carina and cavoatrial junction. For categorical variables, numbers and percentages were presented. For each classification task, the AUC and 95 % Confidence interval (95 % CI) with 1000 bootstrap iterations were performed using Scikit-learn v1.2 [23]. The AUCs within the same class group, such as the ETT group, which consists of “ETT_Normal”, “ETT_Abnormal” and “ETT_Borderline”, were averaged and presented for both the AI-labeled group and resident-labeled group. A P-value of <0.05 and a non-overlapping 95 % CI were considered statistically significant.

## 3 Results

### 3.1 Local dataset

The study included 303 patients with a median age of 71 ± 24 years and approximately equal proportions of males and females. Most of the patients (22.1 %) are from Internal Medicine ICU, followed by General Surgery ICU (12.2 %) and Cardio-thoracic Surgery ICU (11.2 %) with the most prevalent tubes and lines being ETTs (n=178) and ECG leads (n=151), as shown in Table 2.

**Table 2:** Patient characteristics in local testing dataset.

Demographics	Total (n=303)
Age, years; median (IQR) Sex, n (%) Male Female	71.0 (24) 150 (49.5) 153 (50.5)
Intensive Care Units, n (%)	
Internal Medicine General Surgery Cardio-Thoracic Surgery Respiratory Care Unit Trauma Stroke Intermediate Cardiac Care Unit Cardiac Care Unit Neurosurgery COVID	67 (22.1) 37 (12.2) 34 (11.2) 34 (11.2) 31 (10.2) 30 (9.9) 27 (8.9) 23 (7.6) 13 (4.3) 7 (2.3)
Number of tubes and lines, n (%)	
Endotracheal tubes (n=178) Normal Borderline Abnormal Central venous catheters (n=148) Normal Borderline Abnormal Swan-Ganz Catheter Nasogastric tubes (n=39) Normal Borderline Abnormal Incompletely imaged	140 (78.7) 33 (18.5) 5 (2.8) 98 (66.2) 27 (18.2) 12 (8.1) 11 (7.4) 20 (51.3) 4 (10.3) 2 (5.1) 13 (33.3)
Number of other lines, n (%)	
ECG leads Pacemaker or Implantable cardioverter-defibrillators Defibrillator pads Ventriculoperitoneal shunt ICD and PCD Pericardial drain	151 (49.8) 7 (2.3) 10 (3.3) 5 (1.7) 11 (3.6) 2 (0.6)

### 3.2 AI evaluation

For quality control, the performance of the original model was tested on the external dataset, yielding an overall AUC of 0.967 (95 % CI 0.965 – 0.973), similar to the original paper's AUC of 0.972 – 0.973 [5]. The AUC for ETT classes was 0.987 (95 % CI 0.985 – 0.991); for NGT classes, it was 0.978 (95 % CI 0.973 – 0.992); and for CVC classes, it was 0.940 (95 % CI 0.938 – 0.948). The "CVC_Borderline" class had the lowest AUC of 0.894 (95 % CI 0.888 – 0.910), while "Swan Ganz Catheter Presence" scored the highest with an AUC of 0.998 (95 % CI 0.997 – 1.0), as depicted in Table 3.

**Table 3:** The original model AUC scores for classification tasks on the external dataset.

Tube and line	AUC (95 % CI)
ETT	
Normal	0.994 (0.993–0.997)
Borderline	0.968 (0.964–0.98)
Abnormal	0.998 (0.998–1.0)
Average	0.987 (0.985–0.991)
NGT	
Normal	0.990 (0.988–0.995)
Borderline	0.955 (0.939–0.988)
Abnormal	0.976 (0.965–1.0)
Incompletely Imaged	0.991 (0.990–0.995)
Average	0.978 (0.973–0.992)
CVC	
Normal	0.930 (0.926–0.943)
Borderline	0.894 (0.888–0.91)
Abnormal	0.939 (0.933–0.956)
Swan Ganz Catheter	0.998 (0.997–1.0)
Average	0.940 (0.938–0.948)
Average AUC	0.967 (0.965–0.973)

The performance of the original algorithm and modified algorithm, with and without the rule-based system, were then evaluated on our local dataset of 303 radiographs. The AUC of the original and modified algorithms were comparable, however, after applying the rule-based system, the average AUC for ETT classification tasks improved by 0.15 from the AUC score of 0.71 (95 % CI 0.70 – 0.75) to 0.86 (95 % CI 0.83 – 0.94), as demonstrated in Table 4.

**Table 4:** AUC scores for classification tasks on the local dataset.

Labeler	Endotracheal Tube	Central Venous Catheter	Nasogastric Tube
	AUC (95 % CI)	AUC (95 % CI)	AUC (95 % CI)
Original Model	0.72 (0.69 – 0.83)	0.85 (0.83 – 0.91)	0.54 (0.53 – 0.58)
Modified Algorithm	0.71 (0.70 – 0.75)	0.92 (0.90 – 0.96)	0.59 (0.55 – 0.68)
Modified Algorithm with rule-based system	0.86 (0.83 – 0.94)	0.92 (0.90 – 0.96)	0.59 (0.55 – 0.68)
Resident 1 alone	0.83 (0.81 – 0.86)	0.82 (0.81 – 0.88)	0.61 (0.58 – 0.69)
Resident 1 with Algorithm	0.92 (0.90 – 0.98)	0.92 (0.90 – 0.96)	0.6 (0.55 – 0.68)
Resident 2 alone	0.85(0.82 – 0.91)	0.85 (0.83 – 0.90)	0.6 (0.57 – 0.68)
Resident 2 with Algorithm	0.89 (0.87 – 0.95)	0.92 (0.91 – 0.96)	0.59 (0.55 – 0.68)

### 3.3 Residents vs. algorithm

Table 4 shows that without algorithm assistance, both residents demonstrated no significant difference in performance compared to the algorithm alone for ETT and NGT classification tasks. For the ETT classification task, the AUC for the algorithm was 0.86 (95 % CI 0.83 – 0.94), while Resident 1 achieved an AUC of 0.83 (95 % CI 0.81–0.86) and Resident 2 achieved an AUC of 0.85 (95 % CI 0.82–0.91). For NGT classification tasks, the algorithm had an AUC of 0.59 (95 % CI 0.55–0.68), compared to Resident 1 with an AUC of 0.61 (95 % CI 0.58–0.69) and Resident 2 with an AUC of 0.60 (95 % CI 0.57–0.68). However, for CVC classification tasks, both residents performed worse than the algorithm alone. The algorithm had an AUC of 0.92 (95 % CI 0.90–0.96), while Resident 1 had an AUC of 0.82 (95 % CI 0.81–0.88) and Resident 2 had an AUC of 0.85 (95 % CI 0.83–0.90).

### 3.4 Residents alone vs. residents with the algorithm

With algorithm assistance, Resident 1 showed significantly improved performance on the ETT classification task with an AUC of 0.92 (95 % CI 0.90–0.98) compared to 0.83 (95 % CI 0.81–0.86), and on CVC classification tasks with an AUC of 0.92 (95 % CI 0.90–0.96) compared to 0.82 (95 % CI 0.81–0.88). Resident 2 demonstrated significantly improved performance on the CVC classification tasks with an AUC of 0.92 (95 % CI 0.91–0.96) compared to 0.85 (95 % CI 0.83–0.90), but showed non-significant improvement on the ETT classification tasks with an AUC of 0.89 (95 % CI 0.87–0.95) compared to 0.85 (95 % CI 0.82–0.91), as shown in Table 4.

Table 5 shows the mean absolute errors (MAEs) between the labeled coordinates and the ground truth coordinates for the carina and cavoatrial junction. There were statistically significant improvements in performance for both residents with algorithm assistance compared to those without assistance (all P < 0.01). With algorithm assistance, the MAE for the carina improved from 4.1 (SD 4.0 mm) to 2.1 mm (SD 3.1 mm) for Resident 1 and from 3.9 (SD 4.6 mm) to 2.3 mm (SD 3.4 mm) for Resident 2. For the cavoatrial junction, the MAE improved from 19.9 (SD 10.2 mm) to 11.9 mm (SD 9.2 mm) for Resident 1 and from 15.0 (SD 7.3 mm) to 9.2 mm (SD 6.7 mm) for Resident 2. The interpretation times for Resident 1 and Resident 2 without algorithm assistance were 02:25:02 and 1:27:41 (HH:MM:SS), respectively. When paired with AI, the interpretation times were reduced to 1:27:41 and 0:20:03 (HH:MM:SS), which were reductions of 25.59 % and 77.13 %, respectively.

**Table 5:** Carina and cavoatrial junction mean absolute error, i.e., distance from labeled coordinates to ground truth.

Labeler	Carina (mm; mean, SD)		Difference (mm, 95 % CI)	P-value	Cavoatrial Junction (mm; mean, SD)		Difference (mm, 95 % CI)	P-value
	Without Algorithm	With Algorithm			Without Algorithm	With Algorithm		
Algorithm only	3.5 (3.2)				13.2 (7.6)			
Resident 1	4.1 (4.0)	2.1 (3.1)	−2.0 (−2.1 – −1.48)	<0.01	19.9 (10.2)	11.9 (9.2)	−8.0 (−8.34 – −7.09)	<0.01
Resident 2	3.9 (4.6)	2.3 (3.4)	−1.6 (−1.81 – −1.16)	<0.01	15.0 (7.3)	9.2 (6.7)	−5.8 (−6.06 – −4.96)	<0.01

### 3.5 Effects of other lines and AUC outcomes

The AUCs for radiographs with and without ECG leads are presented in Table 6. The AI showed superior performance on ETT classification tasks for images without ECG leads compared to those with ECG leads, with AUCs of 0.90 (95 % CI 0.88–0.94) and 0.78 (95 % CI 0.77–0.81), respectively. However, there was no statistically significant difference observed for CVC classification tasks, with AUCs of 0.93 (95 % CI 0.91–0.98) and 0.91 (95 % CI 0.89–0.97), respectively. Regarding residents, no statistically significant difference was found in ETT classification tasks for images with and without ECG leads. Resident 1 performed significantly better on CVC classification tasks for images without ECG leads compared to those with ECG leads, with AUCs of 0.95 (95 % CI 0.94–0.98) and 0.90 (95 % CI 0.88–0.94), respectively. The AUC for the NGT classification task cannot be calculated because the result was undefined. Additionally, the number of other tubes and lines obtained was too small for analysis.

**Table 6:** Effects of ECG leads on AUCs of algorithm and residents with and without algorithm assistance.

Labeler	Endotracheal Tube		Central Venous Catheter	
	ECG lead	No ECG leads	ECG lead	No ECG leads
	AUC (95 % CI)	AUC (95 % CI)	AUC (95 % CI)	AUC (95 % CI)
Algorithm	0.78 (0.77 – 0.81)	0.9 (0.88 – 0.94)	0.91 (0.89 – 0.97)	0.93 (0.91 – 0.98)
Resident 1 alone	0.81 (0.79 – 0.85)	0.85 (0.83 – 0.90)	0.90 (0.88 – 0.94)	0.79 (0.77 – 0.85)
Resident 1 with Algorithm	0.89 (0.82 – 0.99)	0.92 (0.90 – 0.97)	0.90 (0.88 – 0.97)	0.95 (0.94 – 0.98)
Resident 2 alone	0.79 (0.73–0.91)	0.88 (0.86 – 0.94)	0.87 (0.84 – 0.94)	0.86 (0.84 – 0.92)
Resident 2 with Algorithm	0.86 (0.79 – 0.97)	0.89 (0.87 – 0.95)	0.91 (0.89 – 0.97)	0.94 (0.92 – 0.98)

## 4 Discussion

In recent years, the use of AI in medical imaging, particularly with DL techniques, has garnered attention. However, translating AI models into clinical practice requires rigorous validation due to challenges such as the limited generalizability and 'Black box' problems [8], [9], [10].

Our study is novel in its focus on testing externally trained DL algorithms, specifically on a local ICU patient dataset, addressing the critical issue of AI generalizability in this vulnerable population. Few studies have specifically tested DL algorithms on local ICU populations, often using in-house curated datasets or being limited to ETT classification [4], [24], [25], [26]. While other studies trained their models on datasets from multiple hospitals [2] or public datasets [3], [11], [12] and tested them on the same source, which did not address the generalizability problem.

Despite using the same labeling criteria, our results showed a significant decrease in AUCs on the local dataset with an AUC of 0.70, compared to the external dataset with an AUC of 0.967, highlighting the limited generalizability problem [9], [10], which could be due to the ICU population's associated conditions, e.g., more opacifications on CXR, increased numbers of tubes and lines, etc., leading to hidden stratification, where rare subpopulations are consistently missed by the model [27]. As freely available ICU databases have primarily been sourced from the USA [28], AI training biases from using public datasets skewed towards Caucasian populations, which is contrasted to our predominantly Southeast Asian population, could also contribute to the problem. Another cause of the discrepancy is the use of transparent NGTs at our institution, reflected in the poor performance of both AI and human participants, with AUCs ranging from 0.54 to 0.61. Using more conspicuous or radiopaque NGTs could mitigate this issue. Tang et al. found similar issues with peripherally inserted central catheters (PICCs) due to their thinner appearance compared to regular CVCs [3].

To address the limited generalizability issue, we modified the model to separate masks by tube and line types, allowing extraction of tip coordinates and calculation of distances between tube tips and anatomical reference points. Integrating these distances into rule-based algorithms on top of the existing DL model significantly improved ETT classification, from an AUC of 0.71–0.86. It is also important to note that this is done without the need for local dataset training. The utilization of spatial correlation between tip position and reference anatomy has also been shown to yield good results in previous studies [2], [4], [24], [25]. However, no improvement was seen in the CVC task in our study, likely because CVC classification cannot rely solely on tip position due to potential issues like azygos vein placement, kinks, loops, or incorrect vessel wall angles [3].

**Fig. 4:**
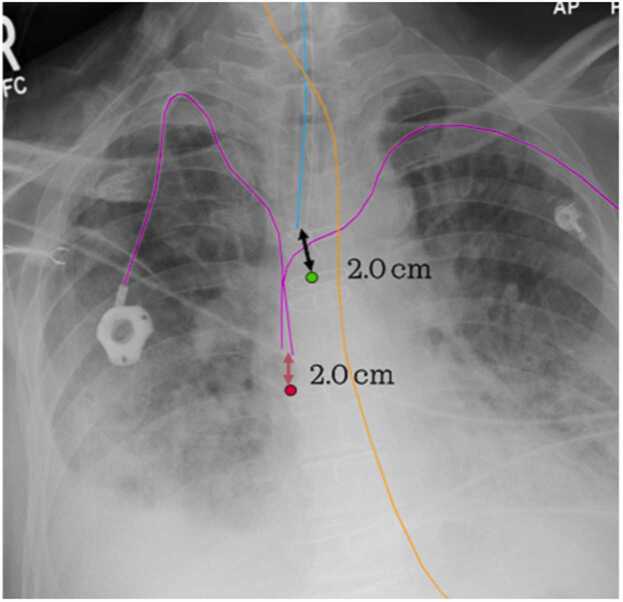
Additional visual aid provided by splitting the channels. There are two CVCs depicted in the image (pink lines), the distance from the CVC tip to the cavoatrial junction (red dot) of 2.0 cm was annotated. The ETT tube (blue line), with its tip 2.0 cm from the carina (green dot), is also depicted. NGT (orange line) is incompletely imaged.

The separation of channels for each tube and line also enables color coding and distance measurement to reference anatomy, aiding in correcting tube and line positioning, as shown in Fig. 4. This helps users understand AI operations and improve accuracy, embodying explainable AI.

Our stratification of patients into subgroups with and without ECG leads revealed a significant improvement in ETT classification performance in the group without ECG leads with an AUC of 0.90, compared to the group with ECG leads with an AUC of 0.78. There was also a slight, but not statistically significant, improvement in CVC classification in the group without ECG leads. The metallic density of ECG leads could be hindering ETT and CVC observation, so we recommend their removal before imaging to potentially enhance AI performance. Training models to accommodate specific hidden stratifications, as demonstrated by a previous study, could also be of help [29].

**Fig. 5:**
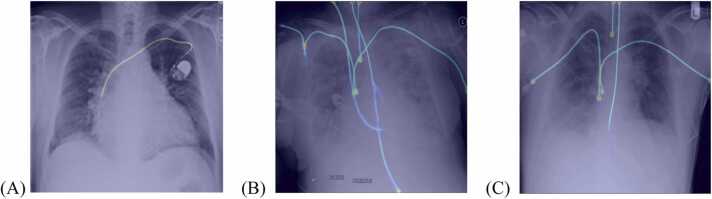
Examples of two result images: (A) False segmentation of CVC. (B) Other catheters interfering with the segmentation of CVC. (C) Especially thin NGT making segmentation of NGT more challenging.

Although not analyzed in this study, we also observed erroneous segmentation of pacemakers as CVCs (Fig. 5A) and other catheters that interfered with the segmentation of CVCs (Fig. 5B). Additionally, in some images the thin NGTs were also incompletely segmented due to obscuration by upper abdominal soft tissue density (Fig. 5C). Even though this didn't confuse the classifier, it suggests that the CLiP dataset, with 9083 segmentation annotations, may not be sufficient.

Lastly, we also included senior radiology residents as inexperienced interpreters in our study. Results showed that AI assistance improved their performance in both tube and line position classification tasks and in labeling carina and cavoatrial junctions. This underscores the potential use of AI in radiology training [30].

There are some limitations to this study. First, the scale of this study, namely, the number of cases, residents, and radiologists involved in this study, is rather small. The limited scale only allowed for the analysis of the ECG leads effects on AI interpretation but not the effects of other tubes and lines, such as chest tubes, percutaneous drainage, ventriculoperitoneal shunts, etc. Secondly, the model was not trained exactly the same way as the original model, due to reasons mentioned above, the AUCs score on control were not significantly lower than the original model. Thirdly, the algorithm itself was not designed to identify multiple CVC lines simultaneously, both in the segmentation and classification steps. Hence, in cases with multiple CVC lines, only the ones closest to the cavoatrial junction were used to calculate the MAEs. Additionally, labeling on the CVAT program may not reflect the time spent on actual image interpretation, as residents needed to record the input into the program, whereas in real life, the interpretation process may end with a single glance at the image. There may also be a bias towards the AI-assisted groups spending less time on interpretation since the images were already labeled on CVAT program by AI, which in turn decreased the number of clicks the residents needed to perform. (Fig. 3 A). We have tried to minimize this bias by automatically placing the coordinates of the cavoatrial junction and carina at the center of the images for the unassisted group (Fig. 3B). Future research should focus on addressing these limitations and improving the generalizability of the model, particularly in the critical care context. One area to explore is the use of metadata, such as the real-world distance between coordinates, to mitigate these limitations. It is also important to use a dataset that includes a diverse population from different geographic and demographic backgrounds to reduce generalizability issues. Furthermore, increasing the size of the dataset and conducting stratification analysis on other types of medical tubes and lines, such as chest tubes and percutaneous drainage, could help to understand the causes of generalizability issues in AI interpretation for ICU patients.

## 5 Conclusion

Externally trained model on general patient radiographs, showed limited generalizability on local ICU dataset. Therefore, it is imperative to evaluate the performance of externally trained AI, such as commercially available AI, for performance on the local population, especially critical care patients. The modified model made use of real-world coordinates to mitigate this problem. However, further research is needed to investigate the cause of this problem and explore potential solutions to improve AI performance.

## Funding

This research (Deep Learning for Tubes and Lines Detection in Critical Illness: Generalizability and Comparison with Residents) did not receive any specific grant from funding agencies in the public, commercial, or not-for-profit sectors. This research received no specific grant from any funding agency.

## Ethical statement

The authors bear full responsibility for all aspects of the work, ensuring that any questions regarding the accuracy or integrity of any part of the work are investigated and resolved. The study received approval from the hospital’s Institutional Review Board (ID: 843/2565(IRB2)), and consent was waived due to its retrospective nature.

## CRediT authorship contribution statement

**Pairash Saiviroonporn:** Writing – review & editing, Supervision, Formal analysis. **Trongtum Tongdee:** Writing – review & editing, Supervision, Project administration. **Pootipong Wongveerasin:** Writing – original draft, Visualization, Validation, Software, Resources, Project administration, Methodology, Investigation, Formal analysis, Data curation, Conceptualization.

## Declaration of Competing Interest

The authors declare that they have no known competing financial interests or personal relationships that could have appeared to influence the work reported in this paper.
